# Clinical practice for unspecified anxiety disorder in primary care

**DOI:** 10.1002/pcn5.118

**Published:** 2023-06-28

**Authors:** Hitoshi Sakurai, Masahiro Takeshima, Ken Inada, Yumi Aoki, Kenya Ie, Morito Kise, Eriko Yoshida, Takashi Tsuboi, Hisashi Yamada, Hikaru Hori, Yasushi Inada, Eiji Shimizu, Kazuo Mishima, Koichiro Watanabe, Yoshikazu Takaesu

**Affiliations:** ^1^ Department of Neuropsychiatry Kyorin University Faculty of Medicine Tokyo Japan; ^2^ Department of Neuropsychiatry Akita University Graduate School of Medicine Akita Japan; ^3^ Department of Psychiatry School of Medicine Kanagawa Japan; ^4^ Department of Psychiatric and Mental Health Nursing St. Luke's International University Tokyo Japan; ^5^ Department of Internal Medicine Division of General Internal Medicine Kanagawa Japan; ^6^ Department of Internal Medicine, Division of General Internal Medicine Kawasaki Municipal Tama Hospital Kanagawa Japan; ^7^ Centre for Family Medicine Development Japanese Health and Welfare Co‐Operative Federation Tokyo Japan; ^8^ Department of General Internal Medicine Kawasaki Kyodo Hospital, Kawasaki Health Cooperative Association Kanagawa Japan; ^9^ Department of Neuropsychiatry Hyogo Medicial University Hyogo Japan; ^10^ Department of Psychiatry, Faculty of Medicine Fukuoka University Fukuoka Japan; ^11^ Medical Corporation YUJIN‐KAI Inada Clinic Osaka Japan; ^12^ Research Center for Child Mental Development Chiba University Chiba Japan; ^13^ Department of Cognitive Behavioral Physiology Graduate School of Medicine Chiba Japan; ^14^ Department of Neuropsychiatry Graduate School of Medicine Okinawa Japan

**Keywords:** anxiolytics, benzodiazepine, primary care, unspecified anxiety disorder

## Abstract

**Aim:**

Clinicians face difficulties in making treatment decisions for unspecified anxiety disorder due to the absence of any treatment guidelines. The objective of this study was to investigate how familiar and how often primary care physicians use pharmacological and nonpharmacological approaches to manage the disorder.

**Methods:**

A survey was conducted among 117 primary care physicians in Japan who were asked to assess the familiarity of using each treatment option for unspecified anxiety disorder on a binary response scale (0 = “unfamiliar,” 1 = “familiar”) and the frequency on a nine‐point Likert scale (1 = “never used,” 9 = “frequently used”).

**Results:**

While several benzodiazepine anxiolytics were familiar to primary care physicians, the frequencies of prescribing them, including alprazolam (4.6 ± 2.6), ethyl loflazepate (3.6 ± 2.4), and clotiazepam (3.5 ± 2.3), were low. In contrast, certain nonpharmacological options, including lifestyle changes (5.4 ± 2.3), coping strategies (5.1 ± 2.7), and psychoeducation for anxiety (5.1 ± 2.7), were more commonly utilized, but to a modest extent. When a benzodiazepine anxiolytic drug failed to be effective, primary care physicians selected the following management strategies to a relatively high degree: differential diagnosis (6.4 ± 2.4), referral to a specialist hospital (5.9 ± 2.5), lifestyle changes (5.2 ± 2.5), and switching to selective serotonin reuptake inhibitor (5.1 ± 2.4).

**Conclusion:**

Primary care physicians exercise caution when prescribing benzodiazepine anxiolytics for unspecified anxiety disorder. Nonpharmacological interventions and switching to SSRI are modestly employed as primary treatment options and alternatives to benzodiazepine anxiolytics. To ensure the safe and effective treatment of unspecified anxiety disorder in primary care, more information should be provided from field experts.

## INTRODUCTION

Anxiety is a common mental health concern in primary care, with prevalence rates varying between 15% and 20% for any current anxiety disorder.^1^, ^2^, ^3^ It affects a broad spectrum of individuals, ranging from those with subthreshold symptoms, which are more commonly reported than diagnosable disorders,^4^, ^5^ to those with specific diagnoses, including generalized anxiety disorder, panic disorder, and social anxiety disorder. Furthermore, there are individuals with unspecified anxiety disorder, characterized by significant anxiety or phobias that do not meet the specific criteria for other anxiety disorders. A cross‐sectional study based on nationally representative data for physician office‐based visits in the United States demonstrated that unspecified anxiety disorder was the primary diagnosis among anxiety disorders, increasing from 50% between 1999 and 2002 to 62% between 2007 and 2010.^6^

Primary care is often the preferred option for individuals seeking treatment for anxiety compared to specialty mental health settings.^7^, ^8^ However, anxiety is frequently undertreated in primary care, leading to patients receiving inadequate pharmacological or psychological treatment, or sometimes no treatment at all.^9^, ^10^ In addition, there is currently a lack of information available on practical management strategies for unspecified anxiety disorder in real‐world settings. The absence of treatment guidelines for this disorder presents a challenge for clinicians in making treatment decisions. The aim of the present study was therefore to investigate the primary care physicians' familiarity and frequency of using pharmacological and nonpharmacological strategies for managing unspecified anxiety disorder.

## METHODS

The task force comprised 13 mental health professionals and primary care providers who identified eight clinical questions concerning the management of unspecified anxiety disorder. For each of these questions, available treatment options were organized according to established treatment guidelines for other anxiety disorders^11^, ^12^, ^13^, ^14^ and clinical practice. Invitations were extended via email to primary care physicians who were affiliated with the Japan Primary Care Association to partake in a questionnaire survey from June 29, 2022, to July 31, 2022. This society has a board certification system in which about 11% of the members have been certified as specialists in this field. Those who provided their consent were asked to assess each treatment option based on two criteria: first, the familiarity with each option on a binary response scale (0 = “unfamiliar,” 1 =  “familiar”), and, second, the frequency on a nine‐point Likert scale (1 =  “never used,” 9 =  “frequently used”). As the options presented in Q5 and Q6 (Table S1) are common, the question regarding familiarity was excluded from these particular questions. The questionnaire included the definition of unspecified anxiety disorder as outlined in the DSM‐5 in their native language (i.e., Japanese). The clinical questions and treatment options are presented in Table S1. The completion of the survey took approximately 15 min. Participation in the survey by the experts was voluntary and without any incentives. Respondents were also asked to indicate their age and gender. The rate of familiarity and the mean value and standard deviation of frequency were calculated for each treatment option. This study was approved by the institutional review board of St. Luke's International University (2021‐604).

## RESULTS

### Participant characteristics

Adequate participation was achieved, with 117 primary care physicians (i.e., members of the Japan Primary Care Association) completing the questionnaire. The mean age of respondents was 47.2 ± 10.3 years. The proportions of male and female respondents were 76.1% and 18.8%, respectively, while 5.1% opted not to provide this information.

### Primary management for unspecified anxiety disorder

More than 80% of the primary care physicians were aware of the use of benzodiazepine anxiolytics including alprazolam (95.7%), ethyl loflazepate (90.5%), lorazepam (87.2%), etizolam (87.2%), clotiazepam (86.3%), diazepam (81.2%), and clonazepam (81.2%) for unspecified anxiety disorder (Table 1). Despite this awareness, the frequencies of prescribing benzodiazepine anxiolytics disorders were low; the most common options were alprazolam, ethyl loflazepate, and clotiazepam, with frequencies of 4.6 ± 2.6, 3.6 ± 2.4, and 3.5 ± 2.3, respectively. In contrast, only three nonpharmacological choices were known by more than 80% of the primary care physicians: lifestyle changes (93.2%), coping strategies (92.3%), and psychoeducation for anxiety (86.3%). However, the frequencies of using these three options were relatively high (lifestyle changes 5.4 ± 2.3, coping strategies 5.1 ± 2.7, and psychoeducation for anxiety 5.1 ± 2.7).

**Table 1:** Primary treatment strategy for unspecified anxiety disorder with more than 80% familiarity.

Treatment strategy	Frequency, mean (SD)
Lifestyle changes	5.4 (2.3)
Coping strategies	5.1 (2.7)
Psychoeducation for anxiety	5.1 (2.7)
Alprazolam	4.6 (2.6)
Ethyl loflazepate	3.6 ((2.4)
Clotiazepam	3.5 (2.3)
Lorazepam	3.4 (2.3)
Etizolam	3.0 (2.3)
Clonazepam	2.2 (1.7)
Diazepam	2.1 (1.6)

*Note*: The options are listed in the order of the mean values of the frequency.

### Management when a benzodiazepine anxiolytic drug is ineffective

When a benzodiazepine anxiolytic drug failed to improve anxious symptoms, several alternative strategies were known to over 80% of the respondents, including switching to a selective serotonin reuptake inhibitor (SSRI) (94.9%), a serotonin and norepinephrine reuptake inhibitor (SNRI) (93.2%), or another benzodiazepine anxiolytic drug (92.3%) and increasing the dose of an anxiolytic drug (92.3%). Among these, the most frequently used options included switching to an SSRI (5.1 ± 2.4), mirtazapine (4.3 ± 2.6), and an SNRI (4.1 ± 2.5) (Table 2). In terms of nonpharmacological management, more than 80% of the primary care physicians were aware of multiple choices, including differential diagnosis (99.1%), referral to a specialist hospital (97.4%), lifestyle changes (94.0%), coping strategies (90.6%), and psychoeducation for anxiety (86.3%). Similarly to primary management, the frequencies of using some nonpharmacological managements, including differential diagnosis (6.4 ± 2.4), referral to a specialist hospital (5.9 ± 2.5), and lifestyle changes (5.2 ± 2.5), were numerically higher than those of any pharmacological strategies, but to a modest extent,.

**Table 2:** Treatment strategy for unspecified anxiety disorder when a benzodiazepine anxiolytic drug fails to improve anxious symptoms with more than 80% familiarity.

Treatment strategy	Frequency, mean (SD)
Differential diagnosis	6.4 (2.4)
Referral to a specialist hospital	5.9 (2.5)
Lifestyle changes	5.2 (2.5)
Switching to SSRI	5.1 (2.4)
Coping strategies	4.9 (2.6)
Psychoeducation for anxiety	4.7 (2.8)
Switching to mirtazapine	4.3 (2.6)
Switching to SNRI	4.1 (2.5)
Switching to another benzodiazepine anxiolytic drug	3.6 (2.2)
Increasing the dose of an anxiolytic drug	3.5 (2.3)
Switching to Kampo	3.2 (2.2)
Combination of two benzodiazepine anxiolytics	2.7 (2.2)
Switching to an antipsychotic drug	2.3 (1.8)

*Note*: The options are listed in the order of the mean values of the frequency.

Abbreviations: SNRI, serotonin and norepinephrine reuptake inhibitor; SSRI, selective serotonin reuptake inhibitor.

### Discontinuation of benzodiazepine anxiolytic drugs

The ratings for all possible options for tapering or discontinuing a benzodiazepine anxiolytic drug following the improvement of anxious symptoms ranged from 3.4 ± 2.3 for the option “immediately after improvement” to 4.6 ± 2.4 for the option “after 1–3 month(s).” Reasons that were deemed acceptable for continuing a benzodiazepine anxiolytic drug included a history of relapsed anxious symptoms (6.3 ± 2.1), anticipation of physical or mental deterioration (6.3 ± 2.1), and patient desire (6.2 ± 2.2). Various strategies were found to be familiar and modestly used when tapering or discontinuing benzodiazepine anxiolytic drugs, including gradual reduction (100.0% and 6.7 ± 2.1, respectively), switching to pro re nata (PRN) (95.7% and 5.4 ± 2.5, respectively), self‐management (94.9% and 4.7 ± 2.4, respectively), and switching to another benzodiazepine anxiolytic drug (92.3% and 4.9 ± 2.3, respectively) (Table 3). Among the pharmacological strategies, switching to an SSRI (94.9% and 5.0 ± 2.7, respectively), an SNRI (91.5% and 4.2 ± 2.6, respectively), and mirtazapine (88.0% and 4.3 ± 2.7, respectively) were familiar and relatively used when tapering or discontinuing a benzodiazepine anxiolytic drug.

**Table 3:** Treatment strategy for tapering or discontinuing a benzodiazepine anxiolytic drug in unspecified anxiety disorder with more than 80% familiarity.

Treatment strategy	Frequency, mean (SD)
Gradual reduction	6.7 (2.1)
Switching to PRN	5.4 (2.5)
Switching to SSRI	5.0 (2.7)
Switching to another benzodiazepine anxiolytic drug	4.9 (2.3)
Lifestyle changes	4.8 (2.6)
Self‐management	4.7 (2.4)
Coping strategies	4.5 (2.6)
Psychoeducation for anxiety	4.5 (2.6)
Switching to mirtazapine	4.3 (2.7)
Switching to SNRI	4.2 (2.6)
Switching to Kampo	4.1 (2.7)
Switching to an antipsychotic drug	2.3 (2.0)

*Note*: The options are listed in the order of the mean values of the frequency.

Abbreviations: PRN, pro re nata; SNRI, serotonin and norepinephrine reuptake inhibitor; SSRI, selective serotonin reuptake inhibitor.

## DISCUSSION

The present study provides insight into the practical management approaches used by primary care physicians for unspecified anxiety disorder. Our findings indicate that primary care physicians exercise caution when prescribing benzodiazepine anxiolytic drugs and resort to alternative management strategies, including fundamental nonpharmacological interventions, to some extent for this condition. This is consistent with expert consensus that benzodiazepine anxiolytics are not recommended as first‐line treatment for unspecified anxiety disorder.^15^

Despite the familiarity, benzodiazepine anxiolytics were not highly prioritized as primary treatment for unspecified anxiety disorder. There was no consistent pattern observed in the duration of action among the familiar and relatively prescribed benzodiazepine anxiolytics for primary treatment. In cases where benzodiazepine anxiolytics were prescribed, physicians tended to discontinue them within a relatively short period once the anxious symptoms improved, unless there were justifiable reasons to continue their use. This is likely because the continuous use of benzodiazepines is associated with various adverse events such as sedation, cognitive impairments, and dependence.^16^ While recent reports indicate that their safety could also be maintained in the long term,^17^, ^18^, ^19^ primary care physicians may limit their use to specified anxiety disorders, including generalized anxiety disorder, panic disorder, and social anxiety disorder, for which benzodiazepine anxiolytics have shown some efficacy.^11^, ^14^ On a different front, there may be some possibility that the respondents of the questionnaire (i.e., members of the Japan Primary Care Association) have better knowledge than general primary care physicians in the treatment of anxiety and the complications associated with benzodiazepines. Considering the prescription rate for anxiolytics in the general population of Japan has remained steady at approximately 2% since 2009,^20^ benzodiazepine anxiolytics may be more commonly utilized for unspecified anxiety disorders in general medical practice than presently reported.

Primary care physicians evaluated fundamental nonpharmacological treatments over benzodiazepine anxiolytics as the initial line of treatment for unspecified anxiety disorder. These treatments included lifestyle changes, coping strategies, and psychoeducation for anxiety, which have demonstrated safety and efficacy in mitigating stress, alleviating anxious symptoms, and promoting favorable psychological outcomes.^21^, ^22^, ^23^, ^24^ It is noteworthy that the respondents of the questionnaire are provided with training on basic nonpharmacological treatments for mental disorders at academic conferences and seminars. Moreover, the specialists of this society are certified based on written examinations, practical examinations, oral examinations, and portfolio evaluations on common clinical practice, including fundamental nonpharmacological interventions and brief psychotherapy for anxiety disorders. However, the frequency to use these strategies was often modest in the present survey, rated 5.4 or less points on a 9‐point scale. Additionally, referral to a specialist hospital followed differential diagnosis as the management primary care physicians conducted when benzodiazepine anxiolytics failed to improve anxious symptoms. To enhance the implementation of these interventions, it is suggested that primary care physicians may need more information from field experts, along with standardized resources, support, and educational programs to alleviate their burden.

When a benzodiazepine anxiolytic drug was ineffective, primary care physicians endorsed switching to an SSRI as a pharmacological alternative at a similar level to nonpharmacological strategies. On tapering or discontinuing a benzodiazepine anxiolytic drug, various pharmacological strategies were employed, including gradual reduction, switching to a PRN, and switching to an SSRI, which were given more preference compared to fundamental nonpharmacological managements. However, the ratings for the frequencies of these treatments were also not high except for gradual reduction, which was rated 6.7 point on a 9‐point scale. These findings indicate that primary care physicians are knowledgeable about alternative benzodiazepine treatments, but not very practiced. It is worth noting that the efficacy of facilitating benzodiazepine discontinuation in chronic benzodiazepine users has only been systematically examined for paroxetine among SSRIs and that there was no significant difference in benzodiazepine discontinuation at the end of intervention between paroxetine and placebo or no intervention.^25^ This lack of evidence should be taken into consideration when determining treatment strategies.

The present study has several limitations that should be considered when interpreting the results. First, the results were based solely on responses to questionnaire surveys completed by primary care physicians, which may not fully reflect actual therapy practices. Second, the lack of adequate information provided for certain clinical questions may have hindered respondents' ability to select appropriate treatment options. The clinical questions concerning alternative interventions of benzodiazepine anxiolytics did not account for the duration of action of these medications. Third, the question used to assess primary strategies for unspecified anxiety disorder did not consider pharmacological treatments other than benzodiazepine anxiolytics. Fourth, the generalizability of our findings may be limited to the Japanese medical community. Finally, the questionnaire survey was conducted exclusively among primary care physicians who were affiliated with the Japan Primary Care Association. Additionally, the proportion of certified specialists from this society among the participants of the present survey were not inquired.

In conclusion, our findings indicate that benzodiazepine anxiolytics are not the primary treatment option for unspecified anxiety disorder according to primary care physicians, despite their familiarity. They are only prescribed for short periods after symptom improvement, if deemed necessary. Fundamental nonpharmacological interventions and switching to SSRIs are preferred for primary treatment and as alternatives to benzodiazepine anxiolytics although the frequencies to use these choices are modest. As unspecified anxiety disorder is often treated in primary care, it is recommended that field experts provide primary care physicians with more information and support to ensure proper treatment for this condition.

## AUTHOR CONTRIBUTIONS

*Acquisition and data analysis, drafting the manuscript*: Hitoshi Sakurai. *Acquisition and data analysis, manuscript revision*: Masahiro Takeshima and Ken Inada. *Acquisition and data analysis*: Yumi Aoki, Kenya Ie, Morito Kise, Eriko Yoshida, Takashi Tsuboi, Hisashi Yamada, Hikaru Hori, Yasushi Inada, Eiji Shimizu, Kazuo Mishima, and Koichiro Watanabe. *Conceptualizaion and study design, acquisition and data analysis*: Yoshikazu Takaesu.

## CONFLICT OF INTEREST STATEMENT

Dr. Sakurai has received grants from the Japan Society for the Promotion of Science KAKENHI (JP22K15755), the Japan Research Foundation for Clinical Pharmacology, and the Takeda Science Foundation, and manuscript and speaker fees from Eisai, Takeda Pharmaceutical, Otsuka Pharmaceutical, Meiji Seika Pharma, Shionogi Pharma, Yoshitomiyakuhin, Sumitomo Pharma, Kyowa Pharmaceutical, and Lundbeck Japan. Dr. Ken Inada has received personal fees/grant support from Eisai, Eli Lilly, Janssen, Meiji Seika Pharma, Mitsubishi Tanabe Pharma, Mochida, MSD, Novartis, Otsuka, Shionogi, Sumitomo Pharma, and Yoshitomiyakuhin in the last three years. Dr. Aoki declares no conflicts of interest. Dr. Takeshima has received speaker's honoraria from Takeda Pharmaceutical, Otsuka Pharmaceutical, Daiichi Sankyo Company, Sumitomo Pharma, Meiji Seika Pharma, Viatris Pharmaceuticals Japan, MSD, Eisai, Ltd., and Yoshitomi Pharmaceutical, and research grants from Otsuka Pharmaceutical, Eisai, Shionogi, and the Japanese Ministry of Health, Labour and Welfare (R3‐21GC1016) outside the submitted work. Dr. Ie has received speaker's honoraria from Eisai and research grants from the Japanese Ministry of Health, Labour, and Welfare (18K15434, 22K15678) and the Japan Agency for Medical Research and Development (21fk0108486h0001) outside the submitted work. Dr. Kise declares no conflicts of interest. Dr. Yoshida declares no conflicts of interest. Dr. Tsuboi has received personal fees from Eisai, Kyowa Pharmaceutical Industry, Meiji Seika Pharma, Mitsubishi Tanabe Pharma, Mochida, MSD, Otsuka, Shionogi, Su‐mitomo Pharma, Takeda Pharmaceutical, Viatris, and Yoshitomiyakuhin within the last three years. Dr. Yamada has received speaker's honoraria from Eisai, Eli Lilly, Kyowa Pharmaceutical Industry, Meiji Seika Pharma, Mitsubishi Tanabe Pharma, Mochida, Otsuka, Sumitomo Pharma, Takeda Pharmaceutical, Viatris, and Yoshitomiyakuhin within the last three years. Dr. Hori has received speaker's honoraria from Eisai, Eli Lilly, Janssen Pharmaceutical, Meiji Seika Pharma, Mitsubishi Tanabe Pharma, MSD, Otsuka Pharmaceutical, Shionogi, Sumitomo Pharma, Viatris, and Takeda Pharmaceutical. Dr. Yasushi Inada has received speaker's honoraria from Eisai, Eli Lilly, Meiji Seika Pharma, MSD, Sumitomo Pharma, Takeda Pharmaceutical, Viatris Pharmaceuticals Japan, and Yoshitomi Pharmaceutical. Dr. Shimizu has received speaker's honoraria from Mochida Pharmaceutical, KYOWA Pharmaceutical Industry, Astellas, Kyorin, and Dainippon Pharma, and research funding from Sumitomo Pharma outside the submitted work. Dr. Mishima has received speaker's honoraria from EISAI, Nobelpharma, Takeda Pharmaceutical, and MSD. Dr. Mishima has received research grants from Eisai, Sumitomo Pharma, Takeda Pharmaceutical, AMED (JP21dk0307103KM), and the Japanese Ministry of Health, Labour and Welfare (19GC1012, 21GC0801). Dr. Watanabe has received manuscript fees or speaker's honoraria from Eisai, Eli Lilly, Janssen Pharmaceutical, Kyowa Pharmaceutical, Lundbeck Japan, Meiji Seika Pharma, Mitsubishi Tanabe Pharma, MSD, Otsuka Pharmaceutical, Pfizer, Shionogi, Sumitomo Pharma, and Takeda Pharmaceutical and received research/grant support from Daiichi Sankyo, Eisai, Meiji Seika Pharma, Mitsubishi Tanabe Pharma, MSD, Otsu‐ka Pharmaceutical, Pfizer, Sumitomo Pharma, and Takeda Pharmaceutical. In addition, Dr. Watanabe is a consultant of Boehringer Ingelheim, Daiichi Sankyo, Eisai, Eli Lilly, Janssen Pharmaceutical, Kyowa Pharmaceutical, Lundbeck Japan, Luye Pharma, Mitsubishi Tanabe Pharma, Otsuka Pharmaceutical, Pfizer, Sumitomo Dainippon Pharma, Taisho Toyama Pharma‐ceutical, and Takeda Pharmaceutical. Dr. Takaesu has received lecture fees from Takeda Pharmaceutical, Sumitomo Dainippon Pharma, Otsuka Pharmaceutical, Meiji Seika Pharma, Kyowa Pharmaceutical, Eisai, MSD, Yoshitomi, and research funding from Otsuka Pharmaceutical, Meiji Seika Pharma, MSD, and Eisai.

## ETHICS APPROVAL STATEMENT

All subjects gave their informed consent for inclusion before they participated in the study. The study protocol was approved by the institutional review board of St. Luke's International University (2021‐604).

## PATIENT CONSENT STATEMENT

N/A.

## CLINICAL TRIAL REGISTRATION

N/A.

## Supporting information

Supporting Information Table S1.

## Data Availability

The data that support the findings of this study are openly available in figshare at https://doi.org/10.6084/m9.figshare.22260397.v1.
