# Supplementary material for: Clinical practice for unspecified anxiety disorder in primary care
Source: PCN Rep. 2023 Jun 28;2(3):e118. doi: 10.1002/pcn5.118 (PMC11114420; doi:10.1002/pcn5.118)
Supplement: Supplementary file 1 — Supporting Information Table S1. [file PCN5-2-e118-s001.docx]

Supplementary Table 1. Questionnaire and responses

In this questionnaire, we inquire about your treatment choices for unspecified anxiety disorder with the following two questions.

(A) Please indicate your familiarity with each option for the given situation.

1. 1

Unfamiliar Familiar

(B) If familiar, please rate how often you would use each option for the given situation.

1 2 3 4 5 6 7 8 9

←Never used　 Frequently used→

Q1) Which benzodiazepine anxiolytics would you prescribe for unspecified anxiety disorder?

| Management | Familiarity, % (n) | Frequency, mean (SD) |
| --- | --- | --- |
| Alprazolam | 95.7 (112) | 4.6 (2.6) |
| Ethyl loflazepate | 90.5 (106) | 3.6 (2.4) |
| Lorazepam | 87.2 (102) | 3.4 (2.3) |
| Etizolam | 87.2 (102) | 3.0 (2.3) |
| Clotiazepam | 86.3 (101) | 3.5 (2.3) |
| Diazepam | 82.9 (97) | 2.1 (1.6) |
| Clonazepam | 81.2 (95) | 2.2 (1.7) |
| Bromazepam | 76.9 (90) | 1.8 (1.3) |
| Chlordiazepoxide | 60.7 (71) | 1.2 (0.8) |
| Cloxazolam | 59.8 (70) | 1.5 (1.1) |

SD, standard deviation

Q2) Which non-pharmacological treatments would you use for unspecified anxiety disorder?

| Management | Familiarity, % (n) | Frequency, mean (SD) |
| --- | --- | --- |
| Lifestyle changes | 93.2 (109) | 5.4 (2.3) |
| Coping strategies | 92.3 (108) | 5.1 (2.7) |
| Psychoeducation for anxiety | 86.3 (101) | 5.1 (2.7) |
| Relaxation techniques | 77.8 (91) | 4.5 (2.8) |
| CBT | 71.8 (84) | 3.3 (2.5) |
| Mindfulness, Attention training | 61.5 (72) | 3.8 (2.8) |

CBT, cognitive behavioral therapy

Q3) Which pharmacological treatments would you use for unspecified anxiety disorder if a benzodiazepine anxiolytic drug did not improve anxious symptoms?

| Management | Familiarity, % (n) | Frequency, mean (SD) |
| --- | --- | --- |
| Switching to SSRI | 94.9 (111) | 5.1 (2.4) |
| Switching to SNRI | 93.2 (109) | 4.1 (2.5) |
| Switching to another benzodiazepine anxiolytic drug | 92.3 (108) | 3.6 (2.2) |
| Increasing the dose of an anxiolytic drug | 92.3 (108) | 3.5 (2.3) |
| Switching to mirtazapine | 88.9 (104) | 4.3 (2.6) |
| Combination of two benzodiazepine anxiolytics | 86.3 (101) | 2.7 (2.2) |
| Switching to an antipsychotic drug | 82.1 (96) | 2.3 (1.8) |
| Switching to Kampo | 81.2 (95) | 3.2 (2.2) |
| Switching to an antihistaminic drug | 77.8 (91) | 2.0 (1.7) |
| Switching to tandospirone | 75.2 (88) | 2.2 (1.7) |
| Switching to an antiepileptic drug | 75.2 (88) | 2.1 (1.8) |

SNRI, serotonin and norepinephrine reuptake inhibitor; SSRI, selective serotonin reuptake inhibitor

Q4) Which management or non-pharmacological treatments would you use for

unspecified anxiety disorder if a benzodiazepine anxiolytic drug did not improve anxious

symptoms?

| Management | Familiarity, % (n) | Frequency, mean (SD) |
| --- | --- | --- |
| Differential diagnosis | 99.1 (116) | 6.4 (2.4) |
| Referral to a specialist hospital | 97.4 (114) | 5.9 (2.5) |
| Lifestyle changes | 94.0 (110) | 5.2 (2.5) |
| Coping strategies | 90.6 (106) | 4.9 (2.6) |
| Psychoeducation for anxiety | 89.7 (105) | 4.7 (2.8) |
| Relaxation techniques | 79.5 (93) | 3.9 (2.7) |
| CBT | 68.3 (80) | 3.1 (2.4) |
| Mindfulness, attention training | 64.1 (75) | 3.3 (2.6) |

Q5) When would you taper or discontinue a benzodiazepine anxiolytic drug after anxious symptoms improve?

| Management | Frequency, mean (SD) |
| --- | --- |
| After 1-3 month(s) | 4.6 (2.4) |
| After 3-6 months | 4.4 (2.2) |
| After 6-12 months | 4.0 (2.3) |
| After more than 12 months | 3.6 (2.4) |
| Immediately after improvement | 3.4 (2.3) |

Q6) Which of the following factors would you consider excusable reasons to continue a

benzodiazepine anxiolytic drug?

| Management | Frequency, mean (SD) |
| --- | --- |
| History of relapsed anxiety symptoms | 6.3 (2.1) |
| Anticipation of physical or mental deterioration | 6.3 (2.1) |
| Patient desire | 6.2 (2.2) |
| No stabilization of physical or mental states or QOL | 5.6 (2.0) |
| Continuation of monotherapy or low dose | 5.5 (2.3) |
| No reported side-effects | 4.3 (2.4) |

QOL, quality of life

Q7) Which strategy would you use for tapering or discontinuing a

benzodiazepine anxiolytic drug?

| Management | Familiarity, % (n) | Frequency, mean (SD) |
| --- | --- | --- |
| Gradual reduction | 100.0 (117) | 6.7 (2.1) |
| Switching to PRN | 95.7 (112) | 5.4 (2.5) |
| Self-management | 94.9 (111) | 4.7 (2.4) |
| Switching to another benzodiazepine anxiolytic drug | 92.3 (108) | 4.9 (2.3) |
| Lifestyle changes | 90.5 (106) | 4.8 (2.6) |
| Coping strategies | 87.2 (102) | 4.5 (2.6) |
| Psychoeducation for anxiety | 86.3 (101) | 4.5 (2.6) |
| Relaxation techniques | 74.4 (87) | 3.8 (2.7) |
| CBT | 63.2 (74) | 3.1 (2.5) |
| Mindfulness, attention training | 59.8 (70) | 3.3 (2.5) |

Q8) Which category of medications would you choose switching to when tapering or discontinuing a benzodiazepine anxiolytic drug?

| Management | Familiarity, % (n) | Frequency, mean (SD) |
| --- | --- | --- |
| SSRI | 94.9 (111) | 5.0 (2.7) |
| SNRI | 91.5 (107) | 4.2 (2.6) |
| Mirtazapine | 88.0 (103) | 4.3 (2.7) |
| Antipsychotic drug | 82.1 (96) | 2.3 (2.0) |
| Kampo | 80.3 (94) | 4.1 (2.7) |
| Antiepileptic drug | 76.1 (89) | 2.0 (1.8) |
| Antihistaminic drug | 72.6 (85) | 2.1 (1.8) |
| Tandospirone | 71.8 (84) | 2.5 (2.2) |
